# Lack of Survival Benefit with Immunotherapy in Combination with Adjuvant Chemoradiation in Pathologic Stage II-IIIB Non-small Cell Lung Cancer

**DOI:** 10.1245/s10434-025-17766-z

**Published:** 2025-07-17

**Authors:** Natasha Venugopal, Jorge Raul Vazquez-Urrutia, Junjia Zhu, Asato Hashinokuchi, Shinkichi Takamori, Takefumi Komiya

**Affiliations:** 1 Department of Medicine, Penn State Health Milton S. Hershey Medical Center, Hershey, PA USA; 2 Department of Public Health Sciences, Penn State College of Medicine, Hershey, PA USA; 3 Department of Surgery and Science, Graduate School of Medical Sciences, Kyushu University, Fukuoka, Japan; 4 Department of Surgery and Science, Graduate School of Medical Sciences, Kyusyu University, Fukuoka, Japan; 5 Division of Hematology Oncology, Penn State College of Medicine, Hershey, PA USA

**Keywords:** Non-small cell lung cancer, Adjuvant chemotherapy, Radiation, Immunotherapy, Atezolizumab

## Abstract

**Background:**

Currently, atezolizumab and pembrolizumab are standard management for curatively resected stage II-III non-small cell lung cancer (NSCLC) owing to prior studies showing that they improve disease-free survival. However, these studies excluded the planned use of adjuvant radiation therapy. Survival benefit of adding immune checkpoint inhibitor (ICI) in patients treated with adjuvant chemoradiation (CT+RT) has not been fully assessed.

**Methods:**

Using National Cancer Database (NCDB), we identified and, based on therapy received, stratified 4,934 cases involving patients undergoing complete resection with pathologic stage II-IIIB NSCLC who survived at least 1 month without neoadjuvant CT or RT and subsequently received adjuvant chemotherapy. Kaplan-Meier methods and multi-variable Cox regression models were used for survival analysis. Propensity score matching was performed to compare adjuvant CT+RT+ICI vs. CT+RT. A *p*-value of <0.05 was considered statistically significant.

**Results:**

The addition of ICI to adjuvant CT improved overall survival (OS) (2-year OS 90.1% vs. 86%, univariate and multivariate hazard ratios [HRs] 0.72 and 0.66,* p* = 0.0024 and 0.0003, respectively). However, no OS benefit was seen in those who received adjuvant CT+RT (2-year OS 77.8% vs. 76.1%, univariate and multivariate HRs 0.83 and 0.85, *p* = 0.3677 and 0.4369, respectively). Propensity score matching analysis showed similar results (2-year OS 77.8% vs. 79.6%, univariate and multivariate HRs 0.91 and 0.87, *p* = 0.7143 and 0.5868, respectively).

**Conclusions:**

Our retrospective real-world analysis suggests that adjuvant ICIs do not improve survival outcome when combined with adjuvant CT+RT. This result mirrors recent negative trials studying ICI+CT+RT in unresectable stage III NSCLC and limited-stage SCLC. Further investigations are warranted.

**Supplementary Information:**

The online version contains supplementary material available at 10.1245/s10434-025-17766-z.

Stage II-IIIB non-small cell lung cancer (NSCLC) are a significant subset of lung cancer cases, characterized by elevated recurrence rates even following surgical resection, with a 3-year recurrence free survival of 58% and 38%, respectively.^1^ The National Comprehensive Cancer Network (NCCN) guidelines illustrate the importance of adjuvant therapies to enhance survival outcomes for these patients.^2^ Concurrent platinum-chemotherapy with radiation is recommended for patients with inoperable stage II and stage III NSCLC, as well as for those undergoing resection with positive margins to strengthen locoregional control.^2^

The introduction of immune checkpoint inhibitors (ICIs) has revolutionized the treatment landscape of NSCLC, in both early and advanced/metastatic settings. The use of ICI in stage II-III NSCLC have recently been studied in peri-operative settings, establishing new standard of care for early stage NSCLC. Promising data from studies such as IMpower-010, and KEYNOTE-091/PEARLS have driven the incorporation of adjuvant atezolizumab and pembrolizumab in the NCCN guidelines for patients with resected stage II-IIIA NSCLC who exhibit PD-L1 expression of ≥1% following chemotherapy.^3^ The IMpower-010 study showed an apparent improvement on disease-free survival (DFS) with adjuvant atezolizumab. Similarly, the KEYNOTE-091/PEARLS trial showed an improvement of DFS in resected stage IB-IIIA, although with less benefit in stage IIIA, chemotherapy nonreceivers.^3^ However, the use of adjuvant radiotherapy was not evaluated in these studies.

Moreover, some studies have shown the benefit of neoadjuvant immunotherapy in NSCLC. The CheckMate 816 trial showed significantly increased event-free survival in pathologic response in stage IB to IIIA resectable NSCLC when combining nivolumab plus platinum-based chemotherapy.^4^ Furthermore, the KEYNOTE-671 trial showed that neoadjuvant pembrolizumab plus chemotherapy followed by resection with adjuvant pembrolizumab, increased overall and recurrence-free survival, major pathological response, and pathological complete response in stage II, IIIA, or IIIB NSCLC patients.^5^

Notably, the aforementioned studies excluded the use of adjuvant radiation. In the absence of definitive evidence, we aim to study the role of ICI within chemoradiation (CT+RT) treatment strategies for this high-risk population in a large, robust sample.

## Material and Methods

National Cancer Database (NCDB) is a joint project of the Commission on Cancer (CoC) of the American College of Surgeons and the American Cancer Society. The CoC’s NCDB and the hospitals participating in the CoC NCDB are the sources of the deidentified data used herein; however, they have not verified and are not responsible for the statistical validity of the data analysis or the conclusions derived by the authors. The data are considered hospital-based rather than population-based.^6–8^

Patients diagnosed with NSCLC diagnosed in 2021 were analyzed (*n* = 8,235). Only cases from 2021 were included owing to the timing of Food and Drug Administration (FDA) approval and availability. Atezolizumab was approved for adjuvant therapy in October 2021, and pembrolizumab was approved in January 2023.^9^ Therefore, we assume patients involved in this analysis who received an ICI were treated with atezolizumab. Additionally, although the NCDB provided data for cases diagnosed up until 2022, cases diagnosed in 2022 were excluded due to survival data not being available. Cases included pathologic stage (p-stage) II-IIIB NSCLC, per American Joint Committee on Cancer (AJCC) 8th edition, that were completely resected, and had to have survived for at least 1 month without neoadjuvant CT or RT. Patients also had to have received adjuvant chemotherapy during the course of their treatment. Exclusion criteria included survival of less than 30 days, other stages of NSCLC, cases not involving surgical resection, and cases that received neoadjuvant therapy.

After applying initial inclusion and exclusion criteria, eligible patients were then assigned to a stratum of (1) adjuvant CT and (2) adjuvant CT+RT. These were further subdivided according to whether the patients received ICI.

The main outcome variable of this study is overall survival (OS), which is defined as the length of time from the initial diagnosis to death of any causes or last follow-up. Key clinical characteristics were obtained and examined within each stratum. These included age (<70 vs. 70 or older), sex (male vs. female), race (white vs. others), Charlson-Deyo comorbidity (CD) score, laterality (R vs. L/other), histology (adenocarcinoma vs. squamous cell vs. other), number of nodes examined (<10 vs. ≥10), p-stage, and EGFR/ALK. PDL1 score was not available.

### Statistical Methods

Our main analyses were stratified by whether the patient received adjuvant chemotherapy or adjuvant chemoradiation in our study sample. Within each stratum, associations between demographics and use of ICI were examined by using chi-square tests. The survival analysis was performed using Kaplan-Meier methods and Cox proportional hazard regression models. Kaplan-Meier survival curves were generated and compared between ICI groups using log-rank tests. Univariate and multivariable Cox proportional hazard regression models were used to calculate hazard ratios (HRs) and their 95% confidence intervals (CIs) for survival since time of tissue diagnosis, and independent prognostic factors were identified. In order to minimize selection bias and balancing baseline characteristics between groups, propensity score matching (PSM) was performed to compare groups (adjuvant CT+RT+ICI vs. CT+RT) using age, sex, pathologic stage, and histology to match the cases according to the XLSTAT software guideline. The PSM was performed using 1:1 nearest-neighbor matching. Finally, 132 matched patients from each group were included in the survival analysis. All other studies were performed using JMP^®^ 14.0 (SAS Institute Inc., Cary, NC). All statistical tests are two-sided, and *p* < 0.05 was considered statistically significant.

## Results

**Fig. 1:**
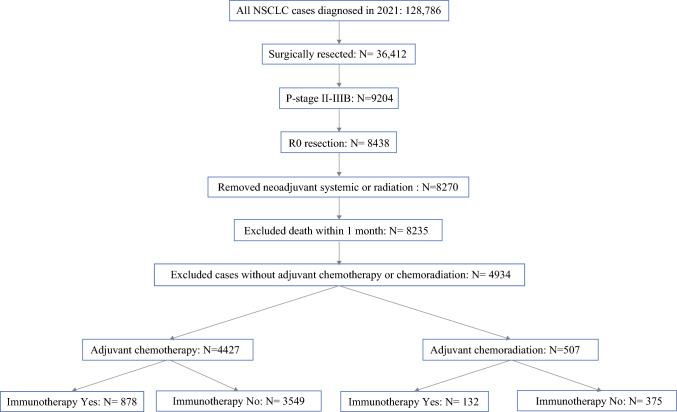
CONSORT diagram detailing the stratification of identified NCDB cases of patients with NSCLC diagnosed in 2021, based on exclusion criteria and division of the criteria-meeting cases into groups based on adjuvant therapy and ICI status. *NCDB* National Cancer Database; *NSCLC* non-small cell lung cancer; *ICI* immune checkpoint inhibitor

Our sample selection process is detailed in Fig. 1. The study reviewed 128,786 patients diagnosed with NSCLC in 2021. After applying exclusion criteria, 8,235 NSCLC p-stage II-IIIB patients were assigned for stratum distribution. Among those patients, 4,427 received adjuvant CT. Patients were divided further into two groups: 878 who received ICI and 3,549 who did not. Of the total 8,235 patients, 507 received adjuvant CT+RT, with 132 of that number also receiving ICI and 375 not receiving ICI.

The clinical characteristics of the patients are shown in Table 1. The proportion of patients receiving ICI was 19.8% in the adjuvant CT group and 26% in the adjuvant CT+RT therapy group. Additionally, white race was most common among the ICI receivers in CT+RT group (*p* < 0.001). Moreover, CT+ICI was associated with negative or unknown EGFR or ALK mutations (*p* < 0.001). Survival analysis demonstrated that younger age, female sex, adenocarcinoma histology, p-stage II, low CD score, positive EGFR/ALK mutation, and ICI were associated with better OS in those who treated with adjuvant chemotherapy by univariate analysis. The consistent result was seen with younger age, female sex, adenocarcinoma, p-stage II, positive EGFR/ALK, and ICI by multivariate analysis. Furthermore, demographic factors, such as CD score 0-1 and positive EGFR/ALK mutations, were associated with better OS in patients who received a combination of CT+RT+ICI, although both those factors were also associated with improved OS in patients who received CT+RT without ICI.

**Table 1:** Clinical characteristics of patients with p-stage II-IIIB NSCLC with or without immunotherapy

Factors		Adjuvant chemotherapy (n = 4,427)			Adjuvant chemoradiation (n = 507)		
Immunotherapy		Immunotherapy			Immunotherapy		
		Yes (n = 878)	No (n = 3,549)	*p*	Yes (n = 132)	No (n = 375)	*p*
Institution							
	Academic	375 (21%)	1386 (79%)	0.047	32 (22%)	112 (78%)	0.217
	Other	503 (19%)	2163 (81%)		100 (28%)	263 (72%)	
Age							
	≥70	356 (20%)	1419 (80%)	0.760	54 (26%)	150 (74%)	0.854
	<70	522 (24%)	2130 (74%)		78 (26%)	225 (74%)	
Sex							
	Male	416 (20%)	1635 (80%)	0.485	60 (25%)	177 (75%)	0.729
	Female	462 (19%)	1914 (81%)		72 (27%)	198 (73%)	
Race							
	White	764 (20%)	2997 (80%)	0.056	117 (27%)	312 (73%)	0.1366
	Other	114 (17%)	552 (83%)		15 (19%)	63 (81%)	
CD score							
	0-1	720 (20%)	2908 (80%)	0.963	107 (26%)	300 (74%)	0.792
	2-3	158 (20%)	641 (80%)		25 (25%)	75 (75%)	
Histology							
	Ad	589 (19%)	2545 (81%)	0.006	100 (27%)	277 (73%)	0.668
	Other	289 (23%)	1004 (77%)		32 (25%)	98 (75%)	
Laterality							
	Right	487 (20%)	2007 (80%)	0.561	71 (26%)	198 (74%)	0.844
	Left/other	391 (20%)	1542 (80%)		61 (26%)	177 (74%)	
No. nodes examined							
	10+	619 (20%)	2498 (80%)	0.946	72 (24%)	223 (76%)	0.324
	<10	259 (20%)	1051 (80%)		60 (28%)	152 (72%)	
p-stage							
	II	502 (18%)	2211 (82%)	0.005	30 (27%)	80 (73%)	0.738
	III	376 (22%)	1338 (78%)		102 (26%)	295 (74%)	
EGFR or ALK							
	Neg/unknown	848 (21%)	3215 (79%)	<0.001	>100 (26%)	>300 (74%)	0.728
	Positive	30 (8%)	334 (92%)		<10*(23%)	<10* (77%)	

*NSCLC* non-small cell lung cancer; *CD* Charlson-Deyo; *Ad* adenocarcinoma; *EGFR* epidermal growth factor receptor; *ALK* anaplastic lymphoma kinase; *Neg*. negative

*Frequencies less than 10 not reported per National Cancer Database guidelines

**Fig. 2:**
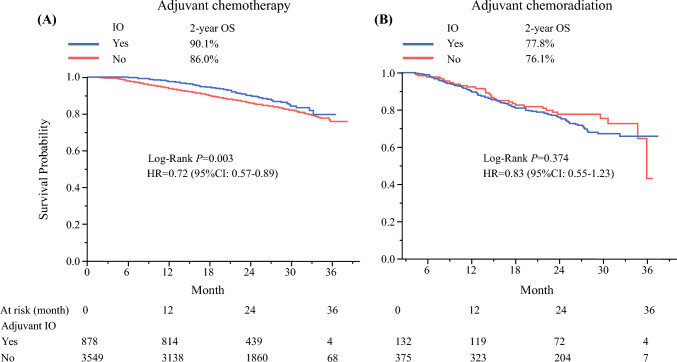
**A** 2-year OS analysis of patients with p-stage II-IIIB NSCLC treated with adjuvant CT and ICI compared to patients treated with adjuvant CT alone. **B** 2-year OS analysis of patients with p-stage II-IIIB NSCLC treated with adjuvant CT+RT plus ICI compared with patients treated with adjuvant CT+RT alone. *OS* overall survival; *NSCLC* non-small cell lung cancer; *CT* chemotherapy; *ICI* immune checkpoint inhibitor; *CT+RT* chemoradiation

Consistent with previous clinical trials, our analysis in Fig. 2 showed that the addition of ICI to adjuvant CT was associated with improved OS, with a 2-year OS of 90.1% with ICI compared with 86% without ICI. Univariate and multivariate analyses further supported these findings (HR 0.72, 0.66, respectively; *p* < 0.001). On the contrary, no OS benefit was observed in those who received adjuvant CT+RT. The 2-year OS was 77.8% with ICI compared with 76.1% without. Univariate and multivariate analyses showed no significant association (HR 0.83, 0.85, respectively; *p* > 0.05) (Table 2). By controlling for potential known confounders through PSM analysis, similar results of no OS benefit with ICI and CT+RT were seen, further supporting the robustness of our findings (Supplemental Fig. 1). The 2-year OS was 77.8% with ICI and 79.6% without ICI. Furthermore, univariate and multivariate analyses showed no association (HR 0.91, 0.87, respectively, *p* > 0.05) (Supplemental Table 2). The analysis was repeated with additional confounders, such as CD score and nodal status, but there were no significant changes noted.

**Table 2:** Univariate and multivariable Cox regression analyses for overall survival in p-stage II-IIIB NSCLC

Factors		Adjuvant chemotherapy				Adjuvant chemoradiation			
		Univariate		Multivariate		Univariate		Multivariate	
		HR (95% CI)	*p*	HR (95% CI)	*p*	HR (95% CI)	*p*	HR (95% CI)	*p*
Institution	Academic/other (Ref) 0.93 (0.79-1.09)	0.93 (0.79-1.1)	0.377	0.84 (0.56-1.22)	0.409	0.81 (0.54-1.19)	0.36		0.287
Age	<70/≥70 (Ref)	0.79 (0.68-0.93)	0.004	0.80 (0.68-0.93)	0.005	0.74 (0.52-1.04)	0.082	0.78 (0.55-1.11)	0.171
Sex	Female/male (Ref)	0.76 (0.65-0.89)	0.001	0.84 (0.72-0.99)	0.039	0.82 (0.58-1.16)	0.266	0.99 (0.7-1.41)	0.973
Race	Other/White (Ref)	0.99 (0.79-1.23)	0.931	1.03 (0.82-1.29)	0.799	0.77 (0.45-1.25)	0.3	0.84 (0.48-1.37)	0.505
CD Score	0-1 / ≥2 (Ref)	0.80 (0.66-0.97)	0.026	0.85 (0.7-1.03)	0.096	0.55 (0.38-0.81)	0.003	0.58 (0.4-0.86)	0.007
Laterality	R/L or other	0.95 (0.81-1.11)	0.505	0.94 (0.8-1.25)	0.441	1.30 (0.92-1.84)	0.141	1.22 (0.86-1.75)	0.26
Histology	Ad/Sq (Ref)	0.61 (0.51-0.72)	<0.0001	0.67 (0.56-0.8)	<0.0001	0.7 (0.47-1.06)	0.093	0.92 (0.61-1.43)	0.706
	Ad/other (Ref)	0.45 (0.33-0.61)	<0.0001	0.48 (0.35-0.65)	<0.0001	0.44 (0.25-0.87)	0.021	0.50 (0.27-1)	0.05
	Sq/other (Ref)	0.74 (0.54-1.02)	0.069	0.72 (0.52-0.99)	0.045	0.63 (0.33-1.3)	0.201	0.54 (0.27-1.14)	0.102
No. nodes examined	<10/10+ (Ref)	0.93 (0.77-1.1)	0.387	0.96 (0.8-1.14)	0.616	0.99 (0.69-1.4)	0.948	1.04 (0.72-1.48)	0.839
p-stage	II/III (Ref)	0.64 (0.55-0.75)	<0.0001	0.64 (0.54-0.74)	<0.0001	1.5 (1.01-2.18)	0.044	1.45 (0.96-2.14)	0.074
EGFR/ALK	P/Neg, unknown (Ref)	0.33 (0.2-0.51)	<0.0001	0.38 (0.23-0.61)	<0.0001	0.11 (0.01-0.49)	0.001	0.14 (0.01-0.62)	0.005
Immunotherapy	Yes/no	0.72 (0.57-0.89)	0.002	0.66 (0.53-0.83)	0.0003	0.83 (0.55-1.23)	0.368	0.85 (0.56-1.27)	0.437

*NSCLC* non-small cell lung cancer; *HR* hazard ratio; *CI* confidence interval; *Ref* reference; *CD* Charlson-Deyo; *R* right; *L* left; *Ad* adenocarcinoma; *Sq* squamous cell carcinoma; *P* positive

For p-stage II-IIIB NSCLC patients receiving adjuvant CT+RT, we successfully matched 132 patients who were also treated with ICI with 132 patients who did not receive ICI, using PSM method. The characteristics after PSM were well balanced and are shown in Supplemental Table 1.

## Discussion

In our analysis of ICI with adjuvant therapy in a large national database, we found that the addition of ICI is not associated with a survival benefit both in p-stage II-IIIB NSCLC patients receiving adjuvant CT+RT but is associated with improved OS in those patients that received only adjuvant chemotherapy alone. This is possibly because of the interaction of CD8 T cells with radiation, which can deplete T-cell populations and, therefore, decrease the efficacy of ICIs.^10^ Radiation may also activate immunosuppressive cells such as regulatory T cells (Tregs) and myeloid-derived suppressor cells (MDSCs), resulting in the inhibition of the function of antigen-presenting cells and dendritic cells.^11–13^ Additionally, toxicity of ICIs could have contributed to the lack of efficacy noted in our and other studies. While the benefit of ICIs has been clearly proven, they have been found to have adverse effects that can cause damage to multiple organs. The toxicities associated with ICIs include but are not limited to pneumonitis, myocarditis, myositis, gastrointestinal symptoms, such as colitis and intractable diarrhea, encephalopathy, hepatic toxicity, renal toxicity, thyroiditis, and rash.

In recent years, the benefits of ICIs for NSCLC have been well-established. However, the management of NSCLC remains challenging, largely due to a lack of studies evaluating the combined efficacy of the available treatment modalities, specifically with the addition of ICI to adjuvant CT+RT. Both the IMpower-010 and KEYNOTE-091/PEARLS studies prohibited adjuvant x-ray radiation therapy, so there has been no data to support the use of ICI after adjuvant CT+RT. Our data suggest that ICI would likely not be a beneficial addition to the treatment regimen of patients with p-stage II-IIIB NSCLC who have already been treated with adjuvant CT+RT, but this finding must be confirmed with further investigation.

Our study analyzed data from the NCDB, the largest clinical cancer database, to evaluate the survival benefits of adding an ICI for p-stage II-IIIB NSCLC patients treated with adjuvant chemotherapy or CT+RT. Our findings demonstrate that combination therapy with ICI plus adjuvant chemotherapy shows survival benefit (2-year OS 90.1% vs. 86%, univariate and multivariate HRs 0.72 and 0.66, *p* = 0.0024 and 0.0003, respectively), but no OS benefit is seen with the addition of ICI to CT+RT (2-year OS 77.8% vs. 76.1%, univariate and multivariate HRs 0.83 and 0.85, *p* = 0.3677 and 0.4369, respectively). These findings are further validated by evidence from several prospective studies. For instance, the Impower-010 and KEYNOTE-091/PEARLS studies, as previously mentioned, also demonstrate the benefit of dual CT and immunotherapy.^3,4^ For patients receiving CT+RT, the PACIFIC2 study is a phase III, multicenter, randomized control trial that is investigating the efficacy of immunotherapy with durvalumab plus CT+RT compared with placebo plus CT+RT.^10^ An update in 2024 demonstrated no improvement of outcomes in the ICI group and noted that approximately 25% of the patients in the ICI group had to have their durvalumab discontinued due to adverse effects.^14^ Conversely, the PACIFIC trial reported improved progression-free survival (PFS) and OS in the durvalumab after CT+RT group and similar safety compared with the placebo after CT+RT group.^15^ The 5-year follow up of this same trial enforced these findings by showing sustained survival benefit in the ICI-CT+RT group, with 42.9% of patients remaining alive after 5 years compared with 33.1% of the placebo group.^16^

Regarding other lung cancers, the NRG LU-005 trial randomized patients with limited stage small cell lung cancer (LS-SCLC) to receive CT+RT alone or combined chemoimmunoradiotherapy followed by consolidative immunotherapy; it failed to achieve its primary endpoint of improved OS.^17^ While cross-trial comparisons are limited, these findings contrast with the survival benefits observed in the ADRIATIC study of immunotherapy after CT+RT in LS-SCLC patients.^18^ Along with the PACIFIC2 trial, this suggests that the addition of immunotherapy during the concurrent CT+RT phase may negate the benefits of subsequent adjuvant immunotherapy, although the underlying mechanisms remain unclear.

It is certainly important to acknowledge some of the limitations inherent in our study. As such, the nonrandomized assignment of patients to treatment groups and the retrospective nature of our analysis introduces inherent biases. To mitigate these, PSM and multivariate analysis were employed; however, biases in comparative effectiveness may still exist based on the choice of treatment.^19^ Furthermore, while the NCDB serves as a valuable resource for cancer-related research, it does not include certain relevant clinical data, such as disease-free survival, specific chemoimmunotherapy regimen, field design, timing relative to immunotherapy, treatment-related toxicities resulting in cessation of immunotherapy, and ECOG performance status, which could influence outcomes. Other potential confounding variables that are not reported in the database could contribute to a worse prognosis for certain patients, leading to them receiving ICIs more often than those with a better prognosis and, therefore, resulting in biased findings. Also, while the database provides OS data for up to 2 years, outcomes for longer periods are not reported, meaning that our study is not able to determine if there were late survival differences. Further studies should have extended follow-up of patients, so these potential differences might be revealed. Additionally, it does not specify that the immunotherapy used was an ICI. However, our team inferred that ICIs would have been the immunotherapy used, given the current research and standard of treatment for patients diagnosed in 2021. Of note, PD-L1 expression is a validated predictive biomarker for ICI efficacy in NSCLC. However, the NCDB does not include PD-L1 status, which limits our ability to perform biomarker-stratified analyses. This remains an important area for future research.

## Conclusions

Our retrospective real-world analysis suggests that adjuvant ICI do not improve survival outcome when combined with adjuvant CT+RT. This result appears to mirror recent negative trials using concurrent use of ICI with CT+RT in unresectable stage III NSCLC (PACIFIC2) and limited-stage SCLC (NRG-LU005). Further investigations are warranted to validate these results and optimize treatment protocols for patients with NSCLC. In the meantime, it would be reasonable to proceed with the concurrent use of ICI with CT+RT, if tolerated, given there is no detrimental effect on OS noted in the current literature.

## Supplementary Information

Below is the link to the electronic supplementary material.

Supplementary file1 (DOCX 16 KB)

Supplementary file2 (DOCX 16 KB)

Supplementary file3 (DOCX 17 KB)

Supplementary file4 (TIFF 126 KB)

## Data Availability

The datasets analyzed during the current study are available via NCDB upon request.
