# Supplementary material for: Lack of Survival Benefit with Immunotherapy in Combination with Adjuvant Chemoradiation in Pathologic Stage II-IIIB Non-small Cell Lung Cancer
Source: Ann Surg Oncol. 2025 Jul 17;32(10):7883–90. doi: 10.1245/s10434-025-17766-z (PMC12454453; doi:10.1245/s10434-025-17766-z)
Supplement: Supplementary file 1 — Supplementary file1 (DOCX 16 KB) [file 10434_2025_17766_MOESM1_ESM.docx]

Supplemental Table 1: Clinical characteristics of p-stage II-IIIB NSCLC patients treated with adjuvant chemoradiation with or without immunotherapy: Propensity-score matched cases

Factors

Immunotherapy

Yes (n=132) No (n=132) P-value

Institution

Academic 32 (45%) 39 (55%) 0.331

Other 100 (52%) 93 (48%)

Age

≥ 70 54 (50%) 54 (50%) 1

< 70 78 (50%) 78 (50%)

Sex

Male 60 (50%) 60 (50%) 1

Female 72 (50%) 72 (50%)

Race

White 117 (52%) 110 (48%) 0.214

Other 15 (41%) 22 (59%)

CD score

0-1 107 (51%) 105 (49%) 0.756

2-3 25 (48%) 27 (52%)

Histology

Ad $\geq$100 (50%) $\geq$100 (50%) 1

Squamous $\geq$10 (50%) $\geq$10 (50%)

Other <10* (50%) <10* (50%)

Laterality

Right 71 (50%) 71 (50%) 1

Left/Other 61 (50%) 61 (50%)

No. of nodes examined

10+ 72 (46%) 86 (54%) 0.078

<10 60 (57%) 46 (43%)

p-stage

II 30 (50%) 30 (50%) 1

III 102 (50%) 102 (50%)

EGFR or ALK

Neg/Unknown $\geq$100 (50%) $\geq$100 (50%) 1

Positive <10* (50%) <10* (50%)

ES, extensive stage; NSCLC, non-small cell lung cancer; CD, Charlson-Deyo; NOS, not otherwise specified; *: Frequencies less than 10 not reported per National Cancer Database guidelines.
