# Supplementary material for: Lack of Survival Benefit with Immunotherapy in Combination with Adjuvant Chemoradiation in Pathologic Stage II-IIIB Non-small Cell Lung Cancer
Source: Ann Surg Oncol. 2025 Jul 17;32(10):7883–90. doi: 10.1245/s10434-025-17766-z (PMC12454453; doi:10.1245/s10434-025-17766-z)
Supplement: Supplementary file 2 — Supplementary file2 (DOCX 16 KB) [file 10434_2025_17766_MOESM2_ESM.docx]

Supplemental Table 2: Univariate and multivariable Cox regression analyses for overall survival in p-stage II-IIIB NSCLC patients treated with adjuvant chemoradiation with or without immunotherapy: propensity-score matching analysis

Immunotherapy Yes/No

N 132/132

Univariate Multivariate

HR (95% CI) HR (95% CI)

P value P value

Institution Academic/Others (Ref) 0.91 (0.51-1.56) 1.10 (0.60-1.92)

P=0.740 P=0.746

Age <70/ 70≤ (Ref) 0.71 (0.44-1.17) 0.83 (0.48-1.43)

P=0.182 P=0.494

Sex Female / Male (Ref) 0.96 (0.59-1.60) 1.35 (0.80-2.31)

P=0.888 P=0.268

Race Others / White (Ref) 0.82 (0.36-1.63) 0.74 (0.32-1.52)

P=0.598 P=0.433

CD Score 0-1 / 2≤ (Ref) 0.35 (0.21-0.59) 0.34 (0.20-0.58)

P=0.0001 P=0.0001

Laterality R/L or other 1.63 (0.99-2.76) 1.60 )0.96-2.75)

P=0.057 P=0.072

Histology Ad/Sq (Ref) 0.85 (0.49-1.57) 1.19 (0.63-2.23)

P=0.586 P=0.593

Ad/Other (Ref) 0.51 (0.16-3.13) 0.31 (0.07-1.40)

P=0.401 P=0.128

Sq/Other (Ref) 0.60 (0.17-3.83) 0.26 (0.05-1.26)

P=0.528 P=0.095

No. of nodes examined <10/10+ (Ref) 1.14 (0.68-1.87) 1.10 (0.65-1.85)

P=0.619 P=0.780

p-stage II/III (Ref) 2.04 (1.20-3.38) 2.18 (1.24-3.75)

P=0.010 P=0.007

EGFR/ALK P/Neg, Unknown (Ref) 6.777e-10 (0.45-0.45) 6.996e-10 (3.39e-317- 0.57)

P=0.004 P=0.999

Immunotherapy Yes/No (Ref) 0.91 (0.56-1.49) 0.87 (0.53-1.44)

P=0.714 P=0.587

NSCLC, non-small cell lung cancer; HR, hazard ratio; CI, confidence interval; Ref, reference; CD, Charlson-Deyo; Ad, adenocarcinoma; Sq, squamous cell carcinoma; EGFR, epidermal growth factor receptor; ALK, anaplastic lymphoma kinase.
