# Supplementary material for: Lack of Survival Benefit with Immunotherapy in Combination with Adjuvant Chemoradiation in Pathologic Stage II-IIIB Non-small Cell Lung Cancer
Source: Ann Surg Oncol. 2025 Jul 17;32(10):7883–90. doi: 10.1245/s10434-025-17766-z (PMC12454453; doi:10.1245/s10434-025-17766-z)
Supplement: Supplementary file 3 — Supplementary file3 (DOCX 17 KB) [file 10434_2025_17766_MOESM3_ESM.docx]

Supplemental Table 3: Additional EGFR/ALK analysis of patients with p-stage II-IIIB NSCLC with or without immunotherapy

Factors Adjuvant chemotherapy (n=4,427) Adjuvant chemoradiation (n=507)

Immunotherapy Immunotherapy

Yes (n=878) No (n=3,549) P-value Yes (n=132) No (n=375) P-value

EGFR or ALK

Positive 30 (8%) 334 (92%) <0.001 NR NR 0.950

Pos (Unk/R) 26 (16%) 138 (84%) NR NR

Negative 351 (25%) 1032 (75%) 37 (26%) 104 (74%)

Unknown 472 (19%) 2046 (81%) 83 (27%) 230 (73%)

EGFR

Positive 25 (8%) 303 (92%) <0.001 NR NR 0.513

Pos (Unk/R) 21 (15%) 119 (85%) NR NR

Negative 469 (24%) 1516 (76%) 55 (24%) 170 (76%)

Unknown 363 (18%) 1611 (82%) 69 (27%) 186 (73%)

ALK

Positive NR NR 0.003 NR NR 0.769

Pos (Unk/R) NR NR NR NR

Negative 369 (22%) 1341 (78%) 47 (27%) 125 (73%)

Unknown 471 (8%) 2155 (92%) 83 (26%) 235 (74%)

NSCLC, non-small cell lung cancer; EGFR, epidermal growth factor receptor; ALK, anaplastic lymphoma kinase; Pos (Unk/R): Positive but unknown or rare; NR: Frequencies less than 10 not reported per National Cancer Database guidelines.
