# Supplementary figures and images for: Lack of Survival Benefit with Immunotherapy in Combination with Adjuvant Chemoradiation in Pathologic Stage II-IIIB Non-small Cell Lung Cancer
Source: Ann Surg Oncol. 2025 Jul 17;32(10):7883–90. doi: 10.1245/s10434-025-17766-z (PMC12454453; doi:10.1245/s10434-025-17766-z)

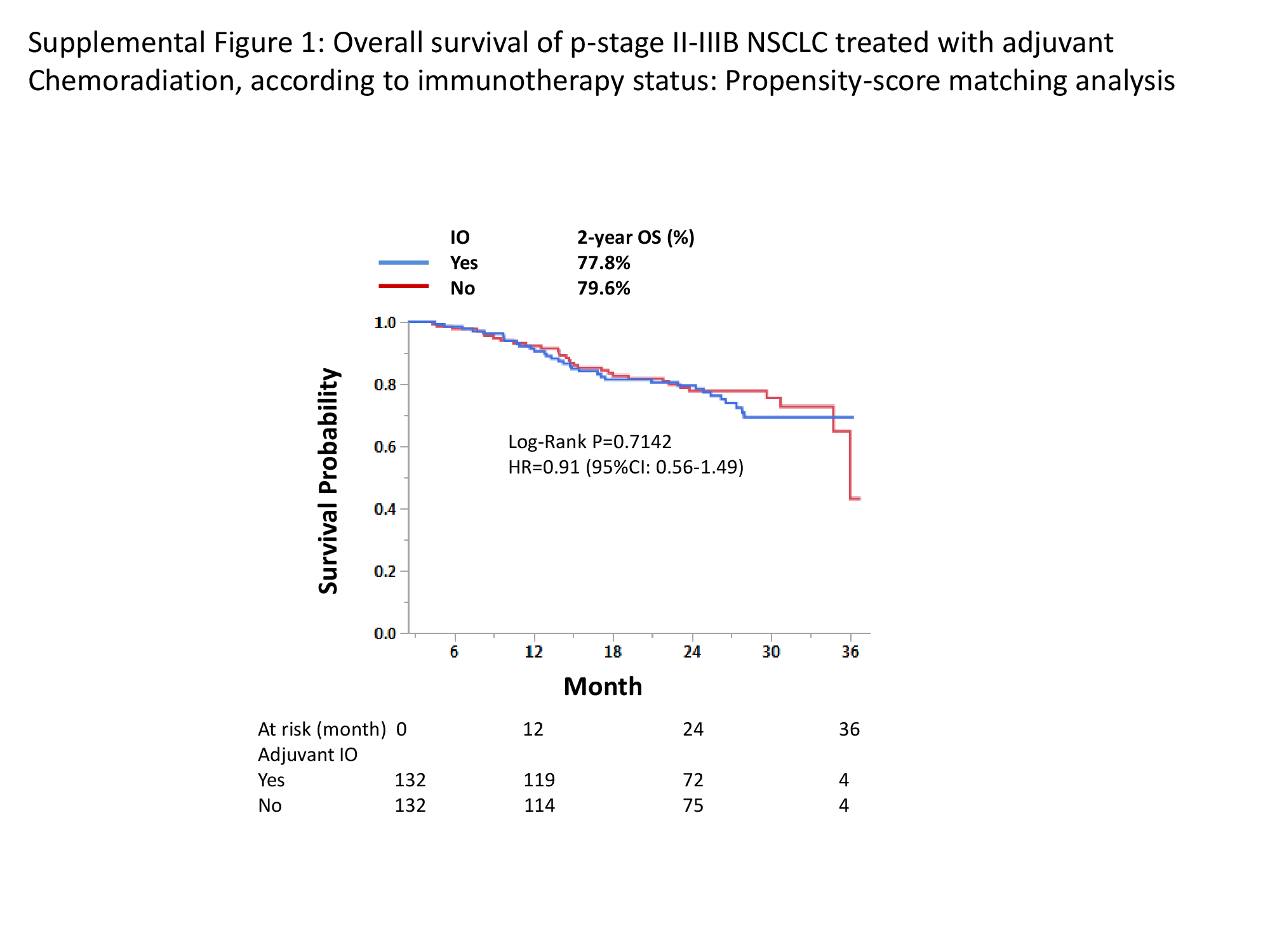

Supplement: Supplementary file 4 — Supplementary file4 (TIFF 126 KB) [file 10434_2025_17766_MOESM4_ESM.tiff]
